# Analysis of atmospheric particles washed away by eyewashes in Indonesia: The second report of the Jakarta study

**DOI:** 10.1016/j.jacig.2025.100527

**Published:** 2025-06-24

**Authors:** Tatsuya Mimura, Willitri A. Sunarya, Kazuhiro Tsuji, Eichi Uchio, Kazumi Fukagawa, Hiroshi Fujishima

**Affiliations:** a Department of Ophthalmology, Teikyo University School of Medicine, Tokyo, Japan; b Department of Ophthalmology, Tsurumi University School of Dental Medicine, Yokohama, Japan; c Rohto Laboratories Indonesia, Jakarta, Indonesia; d Rohto Pharmaceutical Co, Ltd, Kizugawa, Japan; e Department of Ophthalmology, Fukuoka University School of Medicine, Fukuoka, Japan; f Ryogoku Eye Clinic, Tokyo, Japan; g Department of Ophthalmology, Keio University School of Medicine, Tokyo, Japan

**Keywords:** Air pollution, car, eye, eyewash, motorcycle, nose, particle

## Abstract

**Background:**

Airborne particles from pollutants aggravate ocular and nasal symptoms.

**Objective:**

We sought to investigate the effectiveness of eyewashing in removing airborne particles from the eyes and improving ocular and nasal symptoms in Jakarta.

**Methods:**

Healthy volunteers were divided into car (n = 15) and motorcycle (n = 15) commuters. Both eyes were washed twice with commercial eyewash, then the number of particles in the washing solution was calculated using a microscope connected to a smartphone. Ocular and nasal symptoms before and after the first eyewash were scored using a modified Japanese Allergic Conjunctival Disease Quality-of-Life Questionnaire.

**Results:**

The number of particles obtained by eyewashing was significantly higher following the first wash than that following the second wash (36.2 ± 23.2 vs 11.5 ± 10.1, *P* < .001). However, the number of particles did not significantly differ between motorcycle and car commuters for both the first (36.1 ± 23.5 vs 36.3 ± 22.9) and the second washes (13.1 ± 12.4 vs 9.9 ± 6.5). Eyewashing significantly improved total ocular (3.2 ± 3.6 vs 0.9 ± 1.3, *P* = .003) and nasal (1.2 ± 1.8 vs 0.3 ± 0.7, *P* = .017) symptom scores.

**Conclusions:**

Both motorcycle and car commuters have many particles in their eyes. Eyewashing removes particles from the eye and improves subjective symptoms. Therefore, eyewashing may be effective in improving ocular symptoms in countries with severe air pollution.

The Indonesian government plans to relocate its capital from Jakarta to Nusantara, a newly planned city on the island of Borneo, approximately 1200 km away, with the transition beginning in 2024. This is due to the increasing environmental problems in Jakarta. Air pollution in Asia is characterized by particles floating together, including particles in the particulate matter with diameter of less than 2.5 μm (PM_2.5_) size range; Asian dust; and exhaust gases from automobiles, soot, and chemicals from factories.^1^ Several previous studies have suggested that airborne particles may lead to ocular surface inflammation.^2^, ^3^, ^4^, ^5^, ^6^, ^7^ Thus, airborne particles may cause chronic ocular surface irritation and dry eye symptoms. For example, diesel,^8^ PM_2.5_ particles,^9^ and Asian dust^10^, ^11^, ^12^ have been reported to exacerbate allergic conjunctivitis. Various airborne particles in the atmosphere come into contact with the respiratory tract and surface of the eye. In addition to seasonal pollen, many types of particles remain suspended throughout the year, including dust, automobile exhaust, PM_2.5_, and Asian dust.^13^ Indoor allergens such as pet hair and dander, house dust, passive smoking, paint, volatile organic compounds, and sick building syndrome can also trigger ocular inflammation.^14^^,^^15^ These particles can enter the eye and cause allergic conjunctivitis as antigens, or the particles can physically damage the ocular surface barrier.^16^ Alternatively, the chemicals in the particles may induce inflammation of the ocular surface.^1^ Eyewashing may be an effective method for removing these particles from the ocular surface or the conjunctival sac.

Protective eyewear is the most effective countermeasure against exposure of the ocular surface to atmospheric particles. However, eyewashing may be effective when atmospheric particles enter the ocular surface or conjunctival sac. In many countries, cup-type eyewashes are widely used as rinsing solutions for ocular surfaces and conjunctival sacs. Fujishima’s group reported that cup-type eyewashes not only alleviated allergic symptoms in eyes of animals,^17^ but also improved symptoms in a conjunctival allergen challenge study in humans using cedar pollen.^18^

We have also demonstrated that eyewashing effectively treats ocular irritation caused by air pollution.^19^^,^^20^ The effectiveness of eyewashing in washing away airborne particles adhering to conjunctival sacs has not been studied in detail. We previously investigated the effectiveness of over-the-counter eyewash in washing away particles in Jakarta, Indonesia, where air pollution is a significant problem.^21^ Herein, we also investigated the relationship between the number of particles obtained by eyewashing and improvement in subjective symptoms.

## Methods

### Study design

This was a physician-initiated, single-center, evaluator-blinded, parallel, 2-arm, nonrandomized controlled study. All participants received the same eyewashing intervention, and the outcomes were compared between a car commuter group and a motorcycle commuter group. Outcome evaluators were blinded to the participants’ group assignment to minimize assessment bias. This study was conducted in accordance with the ethical principles of the World Medical Association Declaration of Helsinki and the Ethical Guidelines for Medical and Health Research Involving Human Subjects, issued by the Ministry of Education, Culture, Sports, Science and Technology (MEXT), the Ministry of Health, Labour and Welfare (MHLW), and the Ministry of Economy, Trade and Industry (METI) of Japan. Studies involving human participants were approved by the Teikyo University Ethical Review Committee. A series of studies, including this one, was registered as clinical trials in the University Medical Information Network for Clinical Trials (registration number: UMIN000013687).

### Participants

Participants were the same as in our previous study.^21^ The target population was randomly selected from company employees in Jakarta: 15 employees who commuted by motorcycle and 15 who commuted by car. The inclusion and exclusion criteria were the same as described in our previous study.^21^ Age of participants ranged from 24 to 55 years (mean ± SD = 34.2 ± 9.1 years); 10 were men and 20 were women. Both eyes of all participants were included, with 60 eyes of 30 participants subjected to the eyewash test. Informed consent was obtained from all participants included in the study.

### Intervention: Eyewashing method

**Fig 1:**
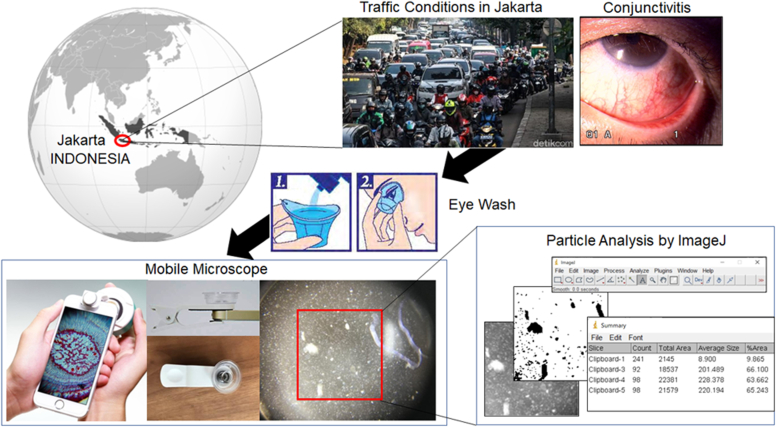
Overview of the study.

Both eyes were washed with ROHTO EyeFlush (Rohto Laboratories Indonesia; Jakarta, Indonesia). Eyewashing was performed according to the procedure described in the ROHTO EyeFlush package insert as shown Fig 1. Washing was performed using an eyewash cup with 5 mL of eyewash solution for 30 seconds as previously reported.^21^

### Subjective symptom score

Both eyes were washed, and subjective symptoms were scored according to a modified Japanese Allergic Conjunctival Disease Quality-of-Life Questionnaire.^19^^,^^20^^,^^22^, ^23^, ^24^

### Measurement of particles in eyewash solution

**Fig 2:**
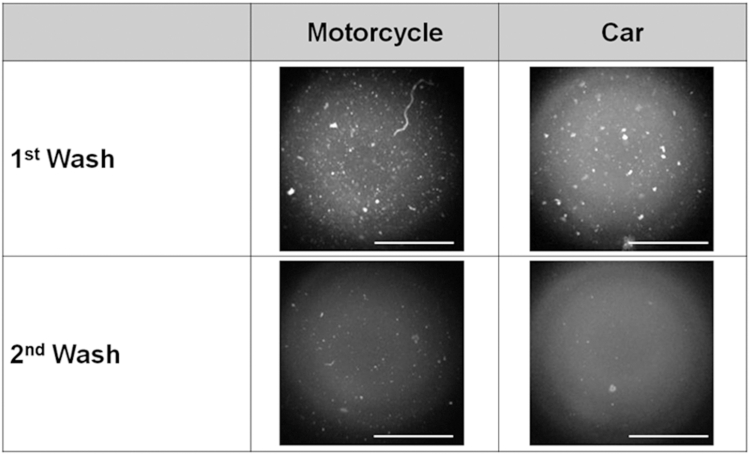
Representative photographs of eyewash solutions collected during the first and second eyewash in motorcycle and car commuters. Bars = 1.0 mm.

Eyewashing was performed twice, and eyewash fluid was collected after the first and second eyewashing as shown Fig 2. Photographs of the total amount of eyewash solution obtained by eyewashing were taken using a solution photographing device (Handy Mobile Microscope Duet; Hi Japan Co, Ltd, Tokyo, Japan) attached to a smartphone. The circular cup that holds the solution has an approximate inner diameter of 25 mm, a height of 10 mm, and a capacity of approximately 5 cm^3^.

The entire volume of the collected eyewash solution was placed in a cup attached to a mobile microscope to ensure that the density of the particles in the solution was uniform. After removing protein components, such as ocular lubricants, the eyewash solution flowed into a cup attached to the mobile microscope. A mobile microscope can acquire laboratory-grade resolution images at up to 1000× magnification. The average number of particles suspended in the center 1.0 mm × 1.0 mm of the solution was determined by analyses of the photographic images using ImageJ analysis software version 1.52a (National Institutes of Health, Bethesda, Md; https://imagej.net/ij/docs/index.html). Although data were collected from both eyes; only data from the right eye were analyzed.

### Statistical analyses

To evaluate the study end points, the 2-tailed unpaired Student *t* test and Wilcoxon signed-rank test were used to compare mean subjective symptom scores and number of particles between the 2 groups, respectively. A 2-tailed paired Student *t* test was used to compare subjective symptom scores and the number of particles before and after eyewashing in the same patient. Data are expressed as mean ± SD. Correlations among the variables were determined by calculating 2-tailed Pearson correlation coefficients and partial correlation coefficients. *P* value ≤ .05 was considered significant. Statistical analyses were performed using SAS version 9.1 (SAS Institute Inc, Cary, NC).

### Availability of data and materials

The datasets used and/or analyzed during the current study are available from the corresponding author on reasonable request.

## Results

### Ocular and nasal subjective symptom score

**Fig 3:**
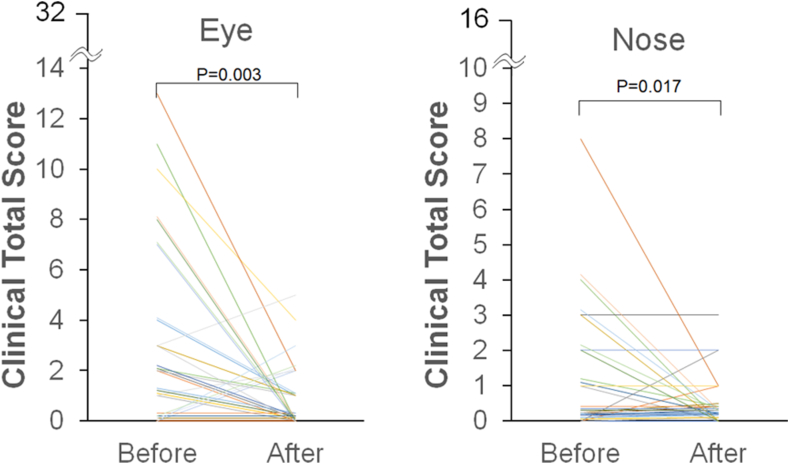
Variation in the total clinical score of eye and nose before and after the first eyewash (n = 30). A 2-tailed paired Student *t* test was used for comparisons before and after eyewashing.

The eyewash intervention did not cause any ocular pain or severe conjunctival hyperemia in any participants, indicating good tolerability and safety. In the analysis of all 30 participants, the total ocular symptom score improved significantly after eyewashing (3.2 ± 3.6 vs 0.9 ± 1.3, *P* = .003) (Fig 3). The total nasal symptom score similarly improved significantly after eyewashing (1.2 ± 1.8 vs 0.3 ± 0.7, *P* = .017) (Fig 3).

**Fig 4:**
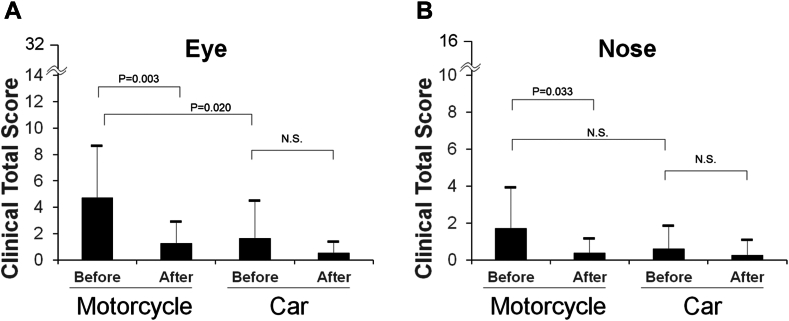
Comparison of total ocular **(A)** and nasal **(B)** subjective symptom scores before and after the first eyewash between motorcycle and car commuters. A 2-tailed paired Student *t* test was used for comparing pre- and post-eyewash scores. A 2-tailed unpaired Student *t* test was used for comparing motorcycle and car commuters. *N.S.,* Not significant.

Data on total ocular subjective symptom scores are shown in Fig 4, *A*. Regarding subjective symptom scores at baseline, the motorcycle group had more severe symptoms than the car group (4.7 ± 3.9 vs 1.7 ± 2.8, *P* = .020). On the other hand, ocular symptom scores after eyewashing were not significantly different between the motorcycle and car groups (1.3 ± 1.6 vs 0.5 ± 0.8, *P* = .131). Comparing the results before and after eyewashing, ocular symptoms improved in the motorcycle group (*P* = .003, paired *t* test; *P* = .015, Wilcoxon signed-rank test), whereas no significant difference was observed in symptom scores before and after eyewashing in the car group (*P* = .147, paired *t* test; *P* = .182, Wilcoxon signed-rank test).

Data on the total subjective nasal symptom scores are shown in Fig 4, *B*. There were no significant differences in subjective nasal symptoms before (1.7 ± 2.2 vs 0.6 ± 1.2, *P* = .092) and after (0.4 ± 0.7 vs 0.3 ± 0.8, *P* = .638) eyewashes between the 2 groups. Nasal symptoms improved in the motorcycle group (*P* = .033), whereas there was no difference in nasal symptom scores before and after eyewashing in the car group (*P* = .389).

### Number of particles in eyewash solution

**Fig 5:**
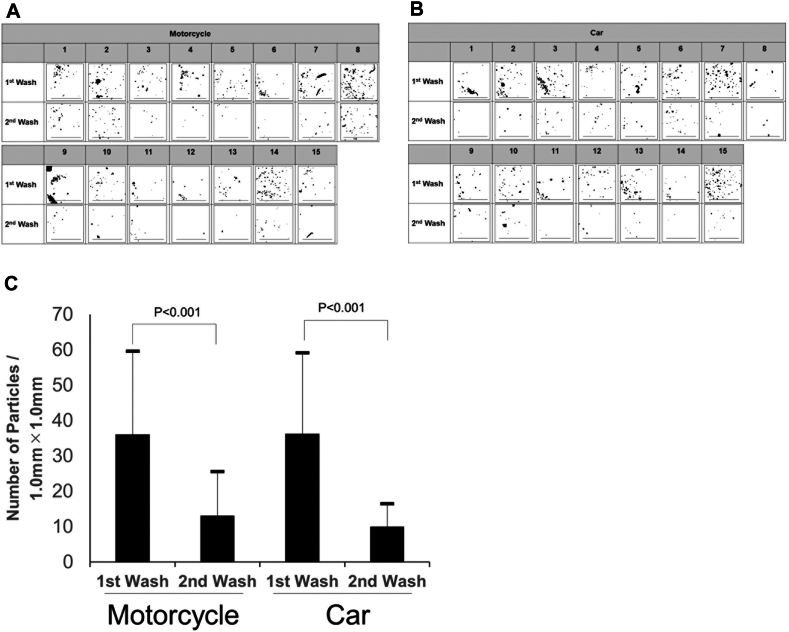
Particles in the wash solution from the first and second eyewashes. **(A)** Photograph of particles in the wash solution for the motorcycle group. Bars = 1.0 mm. **(B)** Photograph of particles in the wash solution for the car group. Bars = 1.0 mm. **(C)** Comparison of the number of particles between the motorcycle and car groups. A 2-tailed paired Student *t* test was used for comparing pre- and post-eyewash scores. A 2-tailed unpaired Student *t* test was used for comparing motorcycle and car commuters.

Photographs of particles obtained by eyewashing are shown in Fig 5, *A* and *B*.

The number of particles/1.0 mm^2^ area obtained by eyewashing was greater in the first eyewash than in the second eyewash for both the motorcycle (36.1 ± 23.5 vs13.1 ± 12.4, *P* < .001) and the car (36.3 ± 22.9 vs 9.9 ± 6.5, *P* < .001) groups.

### Relationship between symptom score and number of particles

**Fig 6:**
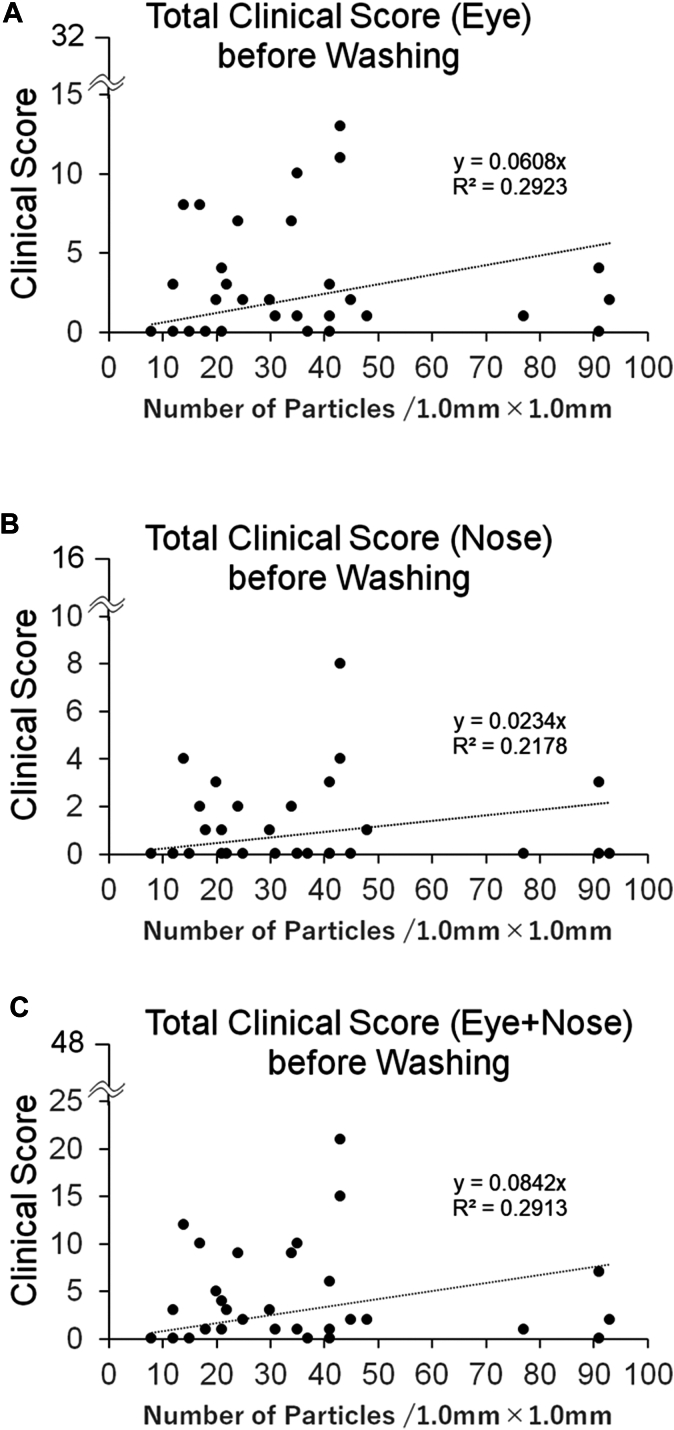
Relationship between total clinical score before eyewash and airborne particles obtained by the first eyewash. **(A)** Eye. **(B)** Nose. **(C)** Sum of the total clinical score for eye and nose.

**Fig 7:**
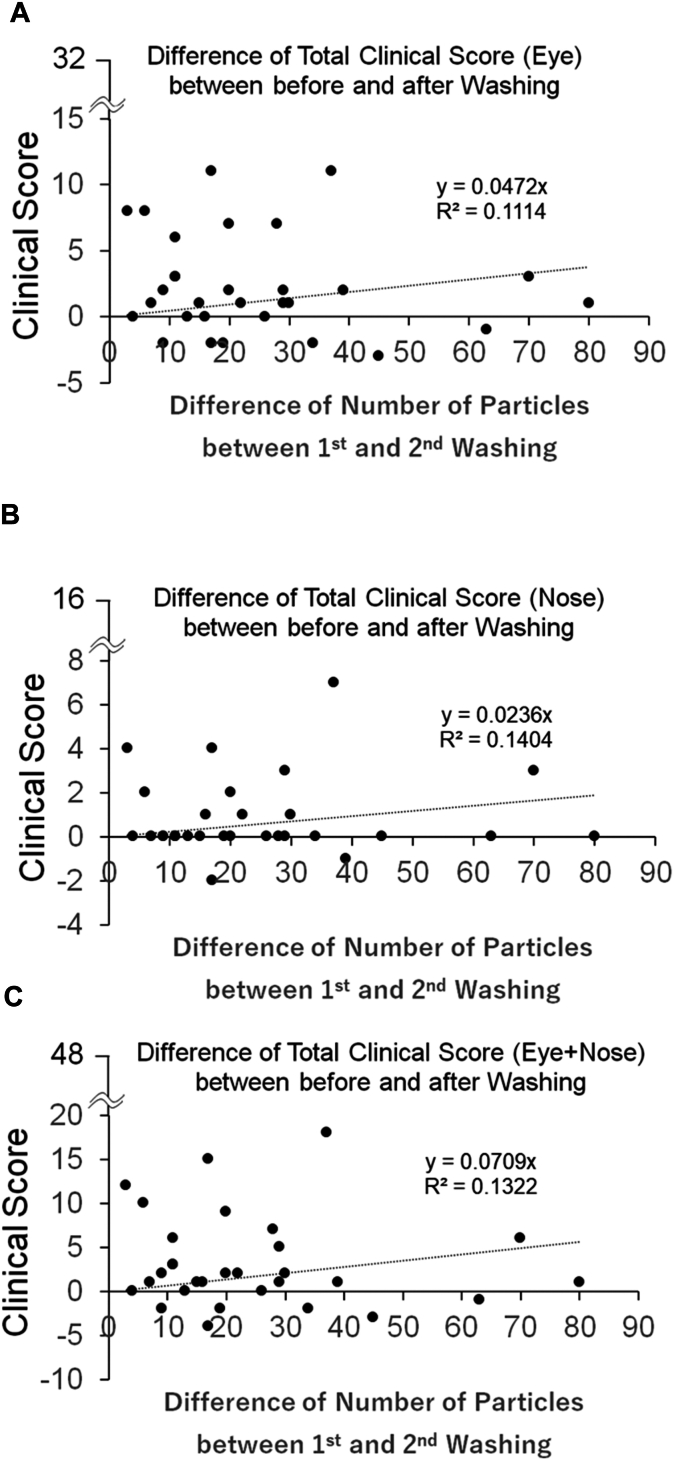
Relationship between the difference in total clinical score before and after eyewash and the difference in airborne particles obtained from the first and second eyewash. **(A)** Eye. **(B)** Nose. **(C)** Sum of the total clinical score for eye and nose.

Fig 6 shows the correlation between the symptom scores before the first eyewashing and the number of particles in the first eyewashing. Fig 7 shows the correlation between the difference in symptom scores before and after the first eyewashing and the difference in particle counts between the first and second eyewashing. Although the symptom scores tended to be higher with the number of particles in the ocular (Figs 6, *A*, and 7, *A*) and nasal (Figs 6, *B*, and 7, *B*) regions than the total score of ocular and nasal regions (Figs 6, *C*, and 7, *C*), no statistically significant correlation was observed between any of the symptom scores and the number of particles.

Table I shows the correlation between the difference in each ocular and nasal symptom score before and after eyewashing and the difference in particle counts between the first and second eyewashing. Only the difference in scores before and after the first eyewashing for foreign body sensation in the eye negatively correlated with the difference between the first and second particles (*r* = −0.42, *P* = .021). No other significant correlations were observed.

**Table I:** Relationship between difference in each ocular and nasal symptom score before and after first eyewash and difference in particle counts between first and second eyewash

Variables	Correlation coefficients		
	*R*	95% CI	*P* value
Ocular symptoms			
Itchy eyes	−0.03	−0.39 to 0.33	.860
Foreign body sensation	−0.42	−0.68 to −0.07	.021
Red eyes	−0.07	−0.42 to 0.30	.713
Watery eyes	−0.31	−0.60 to 0.06	.097
Eye mucus	−0.25	−0.56 to 0.13	.191
Dry eyes	0.21	−0.16 to 0.53	.269
Eye strain	0.05	−0.31 to 0.41	.778
Eye irritation	−0.22	−0.53 to 0.16	.253
Nasal symptoms			
Runny nose	−0.10	−0.45 to 0.27	.594
Sneezing	0.07	−0.29 to 0.42	.694
Stuffy nose	0.18	−0.20 to 0.50	.354
Itchy nose	0.09	−0.28 to 0.44	.638

Correlations were calculated using 2-tailed Pearson product-moment formula.

## Discussion

This study revealed that 1) after commuting to work, motorcycle commuters had more severe conjunctivitis symptoms than car commuters, but there was no difference in rhinitis symptoms, and 2) eyewashing significantly relieved eye symptoms in motorcycle commuters and showed a nonsignificant trend toward symptom relief in car commuters. These results show that eyewashing removes particles from the eye and alleviates subjective ocular symptoms.

First, we looked at the effects of eyewashing on ocular and nasal symptoms. Comparing the baseline symptoms between the motorcycle and car groups, subjective ocular symptoms were more severe for motorcycle commuters than car commuters. This is probably because motorcycle commuters, even though they wear helmets, have their eyes more directly exposed to open air, so their subjective ocular symptoms are more intense than those of car commuters.

Comparing the effects of eyewashing on the motorcycle and car groups, both eye and nasal subjective symptom total scores tended to improve after the first eyewash. Statistical analysis revealed a significant improvement in symptoms in the motorcycle group, whereas the car group exhibited a trend toward improvement, though the difference did not reach statistical significance. Nevertheless, a numerical trend toward symptom relief was observed, suggesting potential individual variability in response. The impact of eyewashing on symptoms was stronger in the motorcycle group than in the car group. This may be because subjective symptoms before eyewashing were higher in the motorcycle group than in the car group, which may have resulted in a more remarkable improvement in subjective symptoms with eyewashing. Symptom scores after eyewashing improved to the same level in the motorcycle and car groups. This suggests that eyewashing alleviated the subjective symptoms of patients.

Second, we looked at the effect of eyewashing on flushing out the particles stored in the conjunctival sac. Particles in the eyewash solution were analyzed after removing prominent protein components, such as ocular lubricants. Therefore, most of the measured particles were of external air component origin. In Jakarta, the number of particles and chemicals in the air on the day of the test was relatively high. The annual average concentrations of airborne substances in Tokyo were 14 μg/m^3^ for particulate matter with diameter of less than 10 μm, 9.8 μg/m^3^ for PM_2.5_, 0.001 ppb for SO_2_, and 0.3 ppm for CO (https://www.metro.tokyo.lg.jp/index.html). These data indicate that Jakarta has approximately 10 times more PM_2.5_ and 60,000 times more SO_2_ than Japan. In Jakarta, many atmospheric particles are expected to enter the atmosphere. Presumably, the number of particles obtained by eyewashing reflects the number of atmospheric particles entering the eye.

The number of particles obtained in the first eyewash was approximately 3 times that in the second wash. Therefore, a single eyewash can reduce the number of particles stored in the eye by approximately one-third of the original amount. These findings indicate that 2 consecutive eyewashes may reduce the number of particles in the conjunctival sac to approximately one-ninth of the baseline level, via a multiplicative effect whereby each eyewash achieves roughly a one-third reduction. Based on these results, 2 eyewashes are recommended for days with heavy air pollution.

Finally, we looked at the correlation between the symptoms and the number of particles. Symptom scores tended to improve after eyewashing with a greater number of particles obtained by washing; however, there was no statistical correlation. This indicates that air pollutants can cause conjunctivitis and rhinitis symptoms regardless of the number of particles, and eyewashing can improve ocular and nasal symptoms.

The motorcycle group had a higher ocular symptom score before the eyewash than the car group. However, the number of particles in the eyewash solution did not differ between the 2 groups. This suggests that, as noted at the beginning of the discussion, the eyes of the motorcycle and car groups received the same amount of atmospheric particles. Still, the motorcycle commuters had more conjunctivitis symptoms owing to direct exposure to the open air.

The correlation between the difference in ocular and nasal symptom scores before and after each eyewash and the difference in particles in the first and second eyewashes were examined. Consequently, only the foreign body sensation correlated negatively with the difference between the first and second particles. This suggests that eyewashing is most effective in reducing the symptoms of a foreign body sensation. The reduction of particles in the conjunctival sac, that is, the reduction of foreign body sensation, may in turn improve other symptoms of red eyes, watery eyes, eye mucus, and eye irritation caused by foreign body reactions to the eye.

In general, commercially available eyewashes are of the cup type. These are designed to be carried on the go and effectively rinse out debris on the spot when it enters the eye. The eyewash did not cause ocular pain or severe hyperemia in any of our participants, indicating that the cup-type eyewash is safe. Several studies have also reported that the cup-type eyewash is safe for the eyes.^17^^,^^18^ and the stability of the epithelial layer, including the oil layer, fluid layer, and mucin of the tear fluid, is vital for the ocular surface barrier.^25^ Therefore, avoiding ocular surface barrier damage owing to excessive eyewashing is essential. Yazu et al^18^ conducted a clinical study using the conjunctival allergen challenge test as a subjective evaluation. These authors proved that eyewashing with cup-type washes not only relieved ocular allergic symptoms, but also did not affect ocular surface mucin or worsen dry eye symptoms.^18^ Pollen, dust, and air pollutants floating in the air as well as debris from eye shadows and eyelash extensions may adhere to the eyelid skin, although they are small and invisible. Therefore, the eyelid should be cleaned using a clean cotton pad when washing the eye with a cup. Avoiding excessive eyewashing and wiping particles from the eyelid skin before rinsing may help minimize the side effects of eyewashing.

This study had several limitations. First, we did not take photographs of the conjunctiva before and after eyewashing; therefore, hyperemia and other symptoms could not be objectively evaluated. Second, comparative studies between Jakarta and other cities are necessary. Third, our study did not account for atopy or allergic sensitization, which could influence symptom responses to eyewashing. Future research should consider these factors to better understand individual variability in treatment effects.

In conclusion, eyewashes cleaned particles from patients’ conjunctival sacs and improved patients’ subjective ocular symptom scores. Cup-type eyewash is a readily available over-the-counter drug and may effectively prevent eye irritation and infection in countries with severe air pollution.

**Key messages**

• Airborne particles accumulate in the eyes of both motorcycle and car commuters, contributing to ocular and nasal discomfort.
• Eyewashing effectively removes a significant number of airborne particles, particularly during the first wash.
• Ocular and nasal symptoms significantly improve after eyewashing, suggesting its potential as a simple and effective intervention.
• Regular eyewashing may be beneficial in regions with severe air pollution, helping to alleviate ocular symptoms and improve overall eye health.

## Disclosure statement

This work was supported in part by a Grant-in-Aid for Scientific Research from the 10.13039/501100001700Ministry of Education, Culture, Sports, Science and Technology of Japan (grant number 20H04347) and an unrestricted investigator-initiated grant from Rohto Pharmaceutical Co, Ltd to T.M.

Disclosure of potential conflict of interest: T. Mimura received a research grant from Rohto Pharmaceutical Co, Ltd. The rest of the authors declare that they have no relevant conflicts of interest.
